# Monks: A Population at Risk for Liver Fluke and Skin-Penetrating Helminths

**DOI:** 10.3390/tropicalmed8030135

**Published:** 2023-02-23

**Authors:** Nuttapon Ekobol, Sirintip Boonjaraspinyo, Atchara Artchayasawat, Thidarut Boonmars

**Affiliations:** 1 Department of Parasitology, Faculty of Medicine, Khon Kaen University, Khon Kaen 40002, Thailand; 2 Cholangiocarcinoma Research Institute, Khon Kaen University, Khon Kaen 40002, Thailand; 3 Department of Community Medicine, Family Medicine and Occupational Medicine, Faculty of Medicine, Khon Kaen University, Khon Kaen 40002, Thailand

**Keywords:** monks, liver fluke, skin-penetrating helminth, raw fish, shoes prohibition, Rules of Discipline

## Abstract

Monks cannot cook received raw meat dishes and should walk barefoot while working. This population lacks both a survey of parasitic infection and a proper prevention and control policy. Five hundred and fourteen monks from the Ubolratana, Ban Haet, and Ban Phai Districts of Kh on Kaen Province were enrolled in this study. A stool container and questionnaire were collected from each study participant. Stool samples were processed by formalin ethyl acetate concentration and agar plate culture techniques. We then analyzed the results and risk factors to demonstrate associations. The prevalence of overall parasites, liver flukes, and skin-penetrating helminths were 28.8%, 11.1%, and 19.3%, respectively. Raw fish dish offerings were associated with opisthorchiasis (OR_crude_ 3.32; 95% CI 1.53–7.20). The risk factors for skin-penetrating helminths were older age (OR_crude_ 5.02; 95% CI 2.2–11.17), being a long-term ordinate (OR_crude_ 3.28; 95% CI 1.15–9.34), smoking (OR_crude_ 2.03; 95% CI 1.23–3.36), and chronic kidney disease with other underlying disease (OR_crude_ 20.7; 95% CI 2.54–190.1). The protective factors for skin-penetrating helminths were secular education above primary education (OR_crude_ 0.41; 95% CI 0.25–0.65) and having received health education about parasitic infection (OR_crude_ 0.47; 95% CI 0.28–0.80). Wearing shoes at times other than alms work does not show a protective effect against skin-penetrating helminths (OR_crude_ 0.86; 95% CI 0.51–1.46). These findings support the recommendation for a strict Rule of Discipline regarding raw meat ingestion and allowing shoes to be worn for protection against skin-penetrating helminths in high-risk situations.

## 1. Introduction

Monks are a population at a unique risk of foodborne parasitic infection and soil-transmitted infection. Monks follow Rules of Discipline [1,2,3], which carry benefits but also risks for parasitic infection. Monks are protected from foodborne parasites by the Discipline of raw meat avoidance. The Buddha allows raw meat and fresh blood in the case of the treatment for demonic possession [1]. Raw meat and some wild animal meats are forbidden [1]. Meat has to be cooked by heat or fire. Water containing small live animals, such as copepods, cannot be used as drinking water [2]. The Lord Buddha allows the use of filter fabric to prevent this drinking water issue [3]. Contrarily, the Rules of Discipline about foods disallow the monks from cooking themselves or ingesting specific meats [1]. Monks cannot further cook raw or undercooked meats after the food is handed to them.

Monks are not only at risk of foodborne parasitic infection, but also of soil-transmitted helminths. Infective eggs are ingested through contaminated foods or dirty hands. Penetrating larvae invade once in contact with the skin [4,5,6,7]. Similar to the risk of foodborne parasites in meat, monks cannot reject or rewash fresh vegetables after receiving them from villagers. The Rules of Discipline regarding allowed and prohibited shoes and wooden shoes for specific types of shoes and specific situations of shoe wearing also hinders skin-penetrating helminth prevention [8]. The Lord Buddha allows some shoes to be worn in specific conditions such as foot ulcers, wounds, or corns, as well as difficulty in traversing a route for pilgrimage [1]. Almost all monks have to beg for alms on bare feet, making them vulnerable to parasites penetrating the skin or foot injuries [1].

The exact prevalence of parasitic infection in monks is not well established. The lack of survey data and the migratory behavior of this population has resulted in a lack of screening, prevention, and control of parasitic infections [9]. The study in Nakhon Pathom Province found a parasite prevalence of 48.6% among 173 monk participants [10]. The most common parasites were hookworms, *Opisthorchis viverrini* (*O. viverrini*), and *Strongyloides stercoralis* (*S. stercoralis*). Another study of parasitic infection in dogs and humans around the temple area with 204 humans (58% were monks or nuns) and 229 dogs found that prevalence of hookworm and strongyloidiasis in humans was 3.4% and 2%, respectively [11]. The most recent study on monks’ health risk of parasitic infection in an urban area with a cement floor environment and some stray dogs and cats in the temple area found a 5.55% prevalence of protozoal infections and did not find helminths in the 36 participants [9].

This study aimed to survey the prevalence of parasitic infection, health behavior risk, and associated factors of parasitic infection in monks from rural areas. The significant factors will be utilized to construct the prevention and control workflow of parasitic infection in monks.

## 2. Materials and Methods

### 2.1. Study Design and Setting

**Figure 1:**
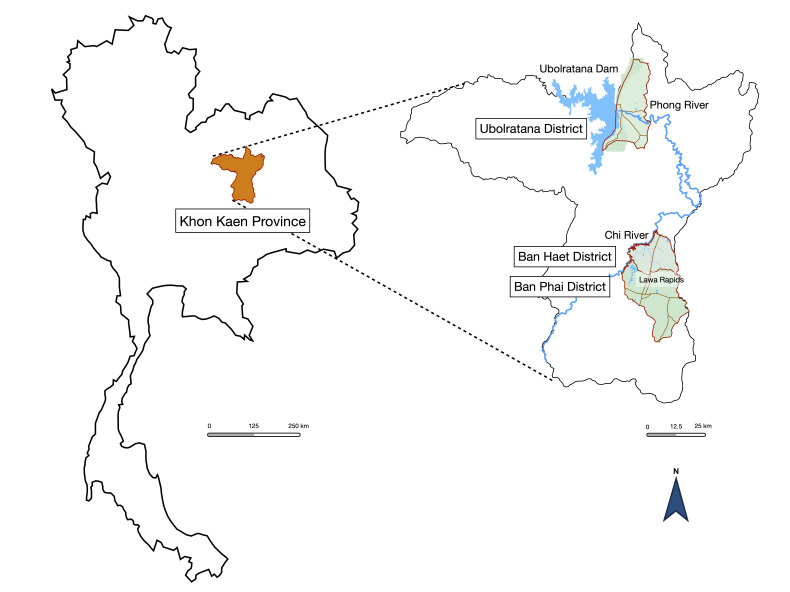
Study areas of Ubolratana District, Ban Haet District, and Ban Phai District of Khon Kaen Province. The association of study areas and water reservoirs was described.

This research was a community-based, cross-sectional study conducted from July 2021 to December 2021. The setting was Ubolratana District, Ban Haet District, and Ban Phai District of Khon Kaen Province, northeastern Thailand (Figure 1). Ban Phai is a large rural area. This district contains the Lawa Rapids and the Chi River in the northwestern part. Ban Haet is above Ban Phai and contains the Lawa Rapids and the Chi River in the southwestern part. Ubolratana District is located near the Ubolratana Dam and the Phong River. These 3 study areas reported the prevalence of opisthorchiasis in the general population in 2004, with 18.1% in the Ubolratana District and 33% in the Ban Phai District [12]. The report from 2020 was 7.7% in Ban Haet District and 8.5% in Ban Phai District [13]. None of the target areas had the highest prevalence of opisthorchiasis cases, but the relationship between communities and water reservoirs created a suitable habitat for liver fluke. According to a northeastern Thailand survey from 2016 to 2017, metacercariae infect 50% of cyprinid fish [14].

### 2.2. Study Population

The monk population in Khon Kaen Province of Thailand is the target population for developing a model of liver fluke prevention and control strategies. Monks that were over 20 years old were included; monks that were on a treatment for parasitic infection or that could not collect stool for examination were excluded. All monk participants were informed about the study objective, benefits, and risks. The consent form was prepared and signed by each participant. The Human Ethics Committee of Khon Kaen University approved all protocols (Reference No. HE641207).

### 2.3. Sample Size Estimation

The sample size was calculated based on the finite single population proportion [15], an assumed proportion of 0.055 from a previous study [9], an acceptable difference of 0.011 (20% of prevalence), and a 95% confidence interval. The total monk population from the 3 study areas was approximately 880 monks (using data from the Khon Kaen Buddhist Office before the rain-retreat survey). The estimated sample size was 575 participants. 

### 2.4. Survey of Parasitic Infection and Data Collection

The self-administered questionnaires and plastic containers were distributed before stool collection. The participants were informed about the stool collection method and completed the questionnaires. Signed consent was given. All questionnaires and stool containers were collected the following morning. The completion of questionnaires was reviewed on site. The fresh morning stool containers were placed in an ice box and transferred to the laboratory room at the Department of Parasitology, Faculty of Medicine, Khon Kaen University. The stool container was opened under the biosafety hood. Three grams of fresh stool was placed on nutrient agar for agar plate culture. The agar plate was incubated at 30 °C for 7 days. The rest of the stool sample was preserved by adding 10% formalin. Stool concentration was determined by the formalin ethyl acetate concentration technique [16,17]. The stool sediments were examined under a compound microscope and the agar plate was examined under a stereomicroscope on day 3, day 5, and day 7 of culture. The positive culture plate by stereomicroscope and the plate at day 7 were rinsed using 10 mL of 10% formalin, and the solution was centrifuged at 2500 rpm for 5 min. The sediment was examined for *S. stercoralis* and hookworm infection under a compound microscope. The agreement of positivity for parasite samples was confirmed by two parasitologists. All infected participants were treated with anti-parasitic drugs and received education about re-infection.

### 2.5. The Questionnaire

The questionnaire for monks consisted of 3 parts. The first part assessed age, ordinate duration, and education (secular and dharma education). The second part assessed health status and history of parasitic infection. The last part assessed health risk behavior for parasitic infection. Age was classified into two groups: <40 years old as young adults and ≥40 years old as middle-aged and senior adults. Secular education was classified as no education to primary education and above primary education. Dharma education was classified as dharma education of advanced level and dharma education lower than advanced level. The duration of monkhood was classified as short-term ordinate (≤1 year) and long-term ordinate (>1 year). Smoking was classified into two groups: no smoking and ex- or current smoking. Alcohol drinking was classified based on history of drinking before monkhood. Anthelmintic drug use was collected as drug use per year and then classified into two groups: no drug use and drug use at least one time per year. The use of non-steroidal anti-inflammatory drugs (NSAIDs) and illegal combined drugs was also collected. Illegal combined drugs are the unknown re-packaging of many types of drugs without drug label by a local shop. This combination drug may contain acetaminophen, 1–3 types of NSAIDs, opioid analgesics, corticosteroids, and some vitamins. Underlying diseases were collected and classified with concern about the effects from multiple-comorbid disease on skin-penetrating helminth infection into chronic kidney disease with other underlying diseases and other underlying disease groups. Offerings of raw meat by the villagers were assessed by the type of meat. Raw meat dishes were reclassified according to the types of raw meat as beef, pork, shrimp, fish, and snail. The self-hygiene questionnaire was composed of a question about drinking water (bucket water, natural water, and tap water), eating a meal with one’s hands, eating freshly cooked food, wearing shoes at other times outside of alms work, toilet use, ground soil defecation, nail hygiene, and hand washing (before eating, after toilet use, after soil contact, and after animal contact). These behaviors were assessed by the frequency of the practice and classified as always practicing and not practicing.

### 2.6. Statistical Analysis

The demographic data were presented as frequencies and percentages. The stool examination result was classified as an infected case by the positive finding of at least one parasite and analyzed for prevalence. Skin-penetrating helminths were classified by positive *S. stercoralis* and/or hookworm findings. The association between risk factors and parasitic infections was analyzed by Pearson’s chi-squared test and binary logistic regression. A *p*-value less than 0.05 indicated statistical significance. The analysis was performed by SPSS version 28.0.1.0 (IBM Corp., Armonk, NY, USA).

## 3. Results

### 3.1. Demographic Characteristics

Five hundred and fourteen monks participated, as shown in Table 1, and the response rate was 89.4%. The mean age and standard deviation were 52.4 ± 17.9 years. Most of the participants (88.7%) were long-term ordinates. Forty-seven percent of participants completed only primary education, but half of the participants completed the Dharma scholar advanced level (53%). Ban Phai District had the highest number of monk participants (62.9%). The smallest participating area was the Ubolratana District (11.3%) due to the district lockdown to prevent the spread of the SARS-CoV-2 virus. Nearly half of the participants had an underlying disease (45.6%). The common underlying diseases were hypertension (12.6%), diabetes (11.5%), and allergy (9.9%). Less than half had previously been educated about parasitic infection, and one third had previously been examined for parasitic infection. Most participants (72.1%) had previous anthelmintic drug use at least one time per year. The experience of receiving raw meat after the ordinate was reported by 79.8% of participants. The most common groups of raw meat that the villagers presented to monks were raw beef dishes (68.8%), raw fish dishes (67.4%), and raw shrimp dishes (57.5%).

### 3.2. Prevalence of Parasitic Infection in Monks

**Figure 2:**
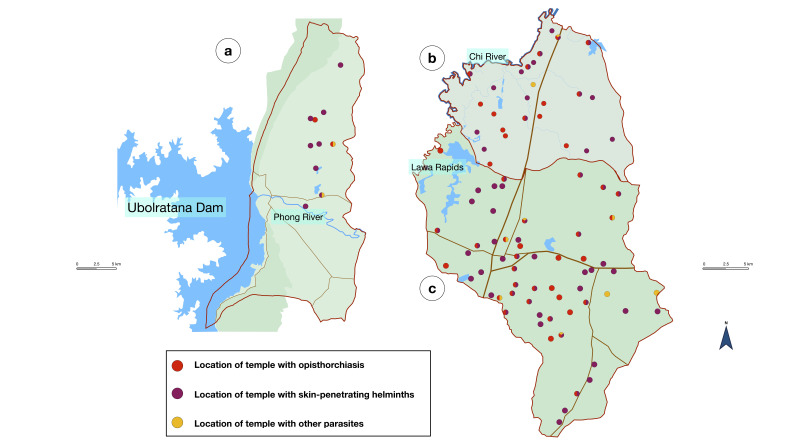
Location of the temples with positive parasites from three districts: (**a**) Ubolratana District; (**b**) Ban Haet District; and (**c**) Ban Phai District. Red color indicates opisthorchiasis, purple color indicates skin-penetrating helminths, and yellow color indicates other parasites.

The overall prevalence of parasitic infection was 28.8% (95% CI 25.0–32.8%), as shown in Table 2. Single infection was 23.3% and multi-infection was 5.5%. Strongyloidiasis was the most common infection, with a prevalence of 15.6%. Liver fluke infection had 11.1% prevalence and hookworm infection had 7% prevalence. Protozoal infection prevalence was very low, from 0.2 to 0.8%. The locations of the temples with prevalent parasites is shown in Figure 2.

### 3.3. Pre-Monkhood Raw Fish Ingestion and Offering Raw Fish Dishes to Monks Associated with Liver Fluke Infection

**Figure 3:**
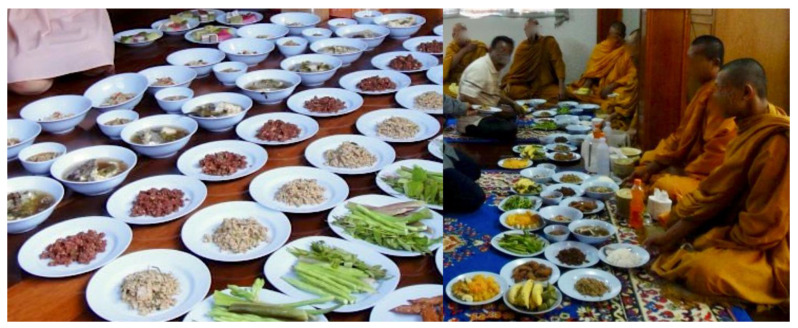
Raw beef salads prepared and offered to monks in a pre-ordinated ceremony.

There were 57 cases of liver fluke-infected monks and almost all cases (95.5%, 42/44, missing data from 13 cases) had a history of raw fish ingestion before monkhood. Eighty-six percent of opisthorchiasis cases reported having been offered raw fish dishes after being ordinated (Figure 3). Being given at least one type of raw fish dish was associated with liver fluke infection in monks (Table 3).

### 3.4. The Forbidden Footwear Rule Assigns a Risk for Skin-Penetrating Helminth Infection to Monks

**Figure 4:**
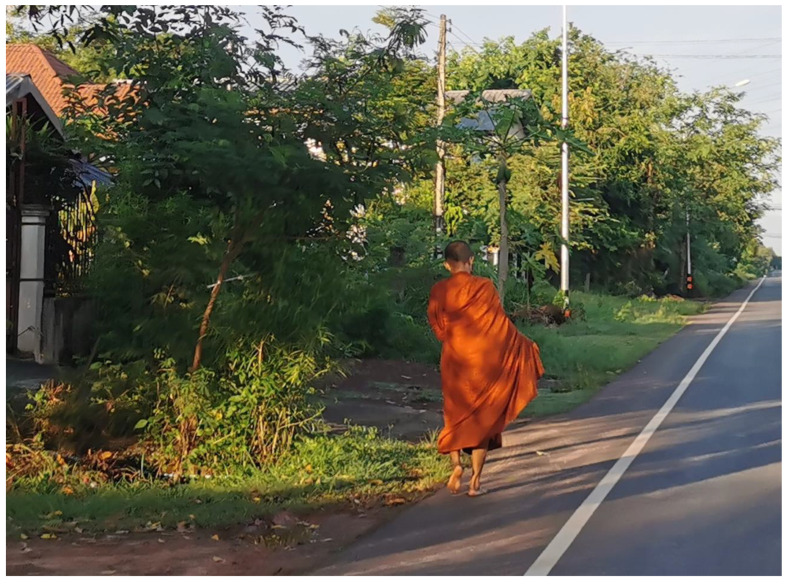
A monk walking barefoot during alms work.

The prevalence of skin-penetrating helminths in monks was 19.3%. The alms work with bare feet is shown in Figure 4. Wearing shoes at a time other than alms work did not show a protective effect from skin-penetrating helminths. The prevalence of infection for always wearing shoes versus not always wearing shoes was 18.6% and 21%, respectively (*p*-value of 0.579).

### 3.5. Defecating on Ground Soil While Performing Off-Site Work Spread Liver Fluke and Skin-Penetrating Helminths

Forty percent of monks defecated on ground soil while performing off-site work. Twenty-eight percent were parasite cases, 8.7% of which were opisthorchiasis cases and 20.1% of which were skin-penetrating helminth cases. Nevertheless, this practice had no statistically significant link with skin-penetrating helminths in monks (*p*-value of 0.940).

### 3.6. Univariate Regression for Opisthorchiasis and Skin-Penetrating Helminth

#### 3.6.1. Opisthorchiasis

Receiving at least one type of raw fish dish was the only significant factor for liver fluke infection in monkhood (OR_crude_ 3.32; 95 %CI 1.53–7.20). Age group, smoking behavior, history of pre-monkhood alcohol drinking, anthelmintic drug use at least one time per year, and education about parasitic infection did not show a difference in opisthorchiasis prevalence (Table 3).

#### 3.6.2. Skin-Penetrating Helminth

The factors that increased the risk of skin-penetrating helminths were older age group (OR_crude_ 5.02; 95% CI 2.26–11.17), long-term ordinate (OR_crude_ 3.28; 95% CI 1.15–9.34), ex-smoking or current smoking (OR_crude_ 2.03; 95% CI 1.20–3.29), and chronic kidney disease with other underlying disease (OR_crude_ 20.7; 95% CI 2.54–190.1) (Table 4).

The factors that decreased risk were secular education above primary education (OR_crude_ 0.41; 95% CI 0.25–0.65) and having received education about parasitic infection (OR_crude_ 0.47; 95% CI 0.28–0.80). The use of NSAIDs or illegal combined drugs, alcohol drinking before monkhood, wearing shoes outside (except during alms work), and anthelmintic drug use at least one time per year did not show any association (Table 4).

## 4. Discussion

This study was the first scientific report on the association between offering traditional raw fish dishes to monks and liver fluke infection, and between the protective effect of shoes on skin-penetrating helminth and shoes prohibiting rules during alms work.

Our study found the prevalence of overall parasitic infection (28.8%) to be lower than the prevalence that was reported in 1989 (48.6%) [10]. Improving health education about parasitic infection and sanitation may lower the risk of infection. However, the monks in our study live in a rural community with a higher risk of parasitic infection by traditional health behavior and a higher chance of soil contact; thus, the prevalence was higher than previous studies in urban monks [9].

Opisthorchiasis was the second most common parasitic infection in monks. The infection and raw fish exposure may continue from secular status to monk status. Almost all opisthorchiasis cases ingested raw fish in traditional foods before being ordinated [18]. This prevalence (11.1%) of liver fluke was lower than the average northeastern Thailand prevalence from 2013 to 2019 of 32.4%, as reported by Thinkhamrop et al. [19], but slightly higher than the previous survey of the general population in 2020: 7.75% from Ban Haet District and 8.48% from Ban Phai District [13]. The distinct prevalence of liver fluke infection in northeastern Thailand changed as a result of the intensive cholangiocarcinoma screening and care program (CASCAP) [20].

Raw beef salad was the most common raw meat dish offered to monks, but the prevalence of taeniasis was low. The low number of observed stool taeniid eggs may have been caused by detached and migratory gravid proglottids that were viable and not ruptured to release the eggs [21]. Another factor is the modern beef production system, which prevents cattle from ingesting taeniid eggs. However, this beef product is only available in the mid-value or premium beef market. Traditional beef markets sell beef products from native cattle reared by a local farm. Cattle feed or graze on communal land, roadside, or leftover byproducts from cultivated areas. This practice increases the risk of bovine cysticercosis [22]. Unfortunately, there are no reports on cattle cysticercosis, the occurrence of cysticercus by beef inspection from slaughterhouses, or market beef product surveys in Thailand [23].

The distribution of raw meat is still practiced in the temple community, and opisthorchiasis-positive monks received raw fish dish offerings. Giving raw fish dishes to monks causes reinfection or new infection. Risk factors include the contamination or unknown cooking of a freshwater fish dish, lack of proper food inspection, and lack of awareness of hygienic food preparation before offering those foods to monks. Insufficient knowledge about parasitic infection and the familiarity and culture of raw meat distribution can influence the offering of raw fish to monks [18,24]. Northeastern Thailand villagers (especially senior adults) offered personally cooked foods to monks and kept the rest to themselves [25]. Raw fish may be present in this case due to their fresh, sweet, soft, and sticky texture, as well as its peak taste [26]. After the monk inspects and takes offered foods, the leftover foods are shared among the participating villagers and taken back to their families. If raw fish dishes are present at this social gathering, it is difficult to avoid distribution to monks and other villagers [18]. Opportunities to address this risk are the cooperation of the Office of Buddhism and civil society to implement two levels to prevent foodborne parasitic infection. The first level involves implementing a training course for villagers to cook safe food for monks and food inspection training for monk attendants. The second level implements education about parasitic infection to newly ordinated monks and those who are health volunteers (the Kilanupatthaka).

Skin-penetrating helminth was the most common soil-transmitted infection in monks. This finding may be caused by their living in the endemic area and their risk for soil contact due to the footwear prohibition rule. This rule assigns easy care to monks in the context of poor socioeconomic communities in the Buddhist era [3]. During this era, shoes were considered luxurious and extravagant, made an annoying sound (wooden shoes in particular), and shoe-wearing monks were accused of having a lustful desire. Monks should not spend time making shoes and should not decorate shoes [1]. This rule is simple for the monk but increases the risk of skin contact with contaminated soil. Our prevalence finding was similar to the prevalence of 15.2% strongyloidiasis in the general population reported in a previous nearby study area in the Nam Phong and Ubolratana Districts of Khon Kaen Province [27]. Moreover, being a long-term ordinate indicated more exposure to contaminated soil and increased the risk of skin-penetrating helminth infection. Alms work results in out-of-temple ground soil contact with the unknown risk of contamination from ground to ground, especially on damp soil and rainy days. Rural villages lack proper water drainage or wastewater irrigation systems. Wastewater soaks the road and sidewalks with eggs and larvae [4]. Wearing shoes for protection outside of alms work does not show a protective effect against this infection.

Footwear provides personal protection against soil-transmitted helminths and other neglected tropical diseases [28]. Wearing shoes is the simplest, most effective, and lowest cost method for preventing infection [29]. Ordinary shoes are not luxury equipment in the current socioeconomic context, and monks do not need to spend additional time making shoes for themselves. Wearing this personal protective equipment should be the right of any person. Additionally, monks are spiritual leaders, and wearing shoes in the correct situations may promote public health policy to prevent skin-penetrating helminths [30]. This topic is critical for encouraging the monks’ practice to be more reasonable and safe while preserving their Rules of the Discipline. Theravada Buddhism has adhered to preserving all the rules in the Basket of the Discipline. Monks should not legislate discipline rules that the Lord Buddha did not legislate and should not withdraw any of the discipline rules [1,31]. Revisions or changes to the rules are nearly impossible, but a new method may be to interpret the rules to be more compatible with modern health practice.

The older age group showed more prevalence of skin-penetrating helminths. This finding is similar to the study of soil-transmitted helminth in the elderly [32]. More advanced age in the sub-group of the elderly showed a higher prevalence of hookworms, *S. stercoralis*, and *Trichuris trichiura*. Adaptive immunity worked differently in the older age group by changing the proportions of T cells, immunoglobulins, and interleukins in a mouse model with filarial worm infection [33]. A decline in the immune response to helminth for both innate and adaptive immunity was also described in another study in the elder age group in combination with the effect of comorbidity [34].

Our study found an association between skin-penetrating helminths and chronic kidney disease. Strongyloidiasis patients had reduced gut microbiota diversity. Shifting of the gut microbial profile towards pathogenic bacteria is associated with a lower GFR or chronic kidney disease [35]. A similar result found in a study of strongyloidiasis and type 2 diabetes with renal complication parameters showed that a significant GFR difference for no diabetes with negative *S. stercoralis* was higher than that for no diabetes with positive *S. stercoralis* [27].

There was no Rule of Discipline to prohibit tobacco smoking. This substance threatens health status but does not have an effect on consciousness and was not banned like alcohol drinking [2]. Tobacco smoking has systemic effects on many organs and shortens the life span of the smoker, especially via cardiovascular events and chronic obstructive pulmonary disease [36,37]. Both current and ex-smoking participants were associated with skin-penetrating helminths infection, but these results are a combination of complex mechanisms. Environmental tobacco smoking or prenatal exposure may affect innate immunity to induce airway inflammation that is hyper-responsive and shifts towards a Th2 response, which may benefit the control of pulmonary helminth infection [38]. Another supporting study shows a correlation between tobacco smoking and decreased worm burden for intestinal helminth infection [39]. On the other hand, immune activation from tobacco smoking is too potent, eventually exhausting and weakening the protective effects of both innate and adaptive immunity [40]. *S. stercoralis* larvae, as well as smoking, contain mechanisms that suppress the pulmonary immune response via shedding cuticle and excretory secretory products [41]. Further investigation of the tobacco smoking effect on skin-penetrating helminths is necessary. Moreover, social influences on cigarette use in monks may result from the pressure from other monks or may be used to relieve stress [42,43].

The monks’ off-site work carries an increased risk for parasitic infection. Villagers may invite monks for community religious rituals. Serving raw meat or raw fish dishes is common in these situations. Pilgrimage is another kind of off-site work, and traveling to a remote area increases the risk of parasitic infection. Limited food choices increase the chance of raw meat being received from local villagers. Pilgrimage to wilderness area benefit skin-penetrating helminth prevention by allowing monks to abstain from the forbidden footwear rule and wear used multilayer shoes. However, remote areas lack restrooms, so monks must defecate on the ground soil, dispersing parasite eggs and larvae to new ground. Yet, there was no association between this practice and infection in monks. Outdoor toilet equipment is required to cut the parasitic life cycle. Screening programs for newly ordinated monks after they return from pilgrimage will benefit parasitic control.

Secular education above primary education showed a lower prevalence of skin-penetrating helminth. There are healthy school projects in Thailand to develop health literacy and enhance the skill of self-hygiene in school children [44]. Incomplete or lack of primary education results in insufficient education about parasitic infection.

Education about parasitic infection is the important factor for primary and secondary prevention. The received intervention package showed additive prevention for the re-infection of *S. stercoralis* [45]. Either way, some monks reported behavioral changes after receiving education about parasitic infection, such as minimizing soil contact and always wearing shoes in the temple area. Some temples modified the area for walking meditation to prevent soil contact by coverage with a plastic sheet.

The limitation of this study was the cross-sectional design with a single stool sampling. A cross-sectional study has limited power to demonstrate direct causes and effects. Monks have migratory behavior and only complied with a single stool sampling. Measuring the exact raw meat ingestion data by monks violates the Rules of the Discipline. Therefore, we only reported the choices of raw meat offerings.

## 5. Conclusions

Monks are a population at high risk for parasitic infection due to traditional raw fish offerings and the Rules of Discipline prohibiting footwear. Strictly applying rules about raw meat ingestion, providing education about parasitic infection, and reinterpreting the footwear prohibition Rule of Discipline to support modern health practices may reduce this risk.

## Figures and Tables

**Table 1:** Sociodemographic characteristics of participants.

Variables	No. of Participants
	*n* (%)
Age (*n* = 511)	
≤20	26 (5.1)
21–40	98 (19.2)
41–60	204 (39.9)
≥61	183 (35.8)
Ordinate (*n* = 450)	
Monkhood ≤ 1 year	51 (11.3)
Monkhood > 1 year	399 (88.7)
Secular education (*n* = 476)	
≤Primary education	226 (47.5)
>Primary education	250 (52.5)
Dharma education (*n* = 438)	
<Dharma scholar advanced level	206 (47.0)
Dharma scholar advanced level	232 (53.0)
Study area (*n* = 512)	
Ubolratana	58 (11.3)
Ban Haet	132 (25.8)
Ban Phai	322 (62.9)
Underlying disease (*n* = 454)	
No	247 (54.4)
Yes	207 (45.6)
Illegal combined drugs (*n* = 433)	
No	394 (91.0)
Yes	39 (9.0)
Smoking (*n* = 480)	
No	196 (40.8)
Ex- or current smoking	284 (59.2)
Alcohol before monkhood (*n* = 411)	
No	196 (47.7)
Yes	215 (52.3)
Education of parasitic infection (*n* = 404)	
Never	216 (53.5)
Received	188 (46.5)
Examination of parasitic infection (*n* = 467)	
No	320 (68.5)
Yes	147 (31.5)
History of parasitic infection (*n* = 408)	
No	307 (75.2)
Yes	101 (24.8)
Anthelmintic drugs per year ≥ 1 time/year (*n* = 480)	
No	134 (27.9)
Yes	346 (72.1)
Offer raw meats to monk (*n* = 485)	
No	98 (20.2)
Yes	387 (79.8)
Raw beef dishes (*n* = 475)	327 (68.8)
Raw fish dishes (*n* = 475)	320 (67.4)
Raw shrimp dishes (*n* = 447)	257 (57.5)
Raw pork dishes (*n* = 462)	256 (55.4)

**Table 2:** Types of parasitic infections in monks.

Parasitic Infection (*n* = 514)	*n*	(%)
Single infection	120	(23.3)
Multi-infection	28	(5.5)
Total infections	148	(28.8)
Helminth		
*Opisthorchis viverrini*	57	(11.1)
*Strongyloides stercoralis*	80	(15.6)
Hookworm	36	(7.0)
*Taenia* spp.	3	(0.6)
*Echinostome* sp.	1	(0.2)
Minute intestinal flukes	1	(0.2)
*Ascaris lumbricoides*	1	(0.2)
Protozoa		
*Giardia lamblia*	4	(0.8)
*Entamoeba histolytica*	1	(0.2)
*Isospora belli*	1	(0.2)
*Entamoeba coli*	3	(0.6)

**Table 3:** The factors associated with *O. viverrini* infection in monks.

Variables	*O. viverrini* Negative	*O. viverrini* Positive	OR_crude_ (95% CI)	*p*-Value
	*n* (%)	*n* (%)		
Age (*n* = 511)				
<40	109 (90.1)	12 (9.9)	1	
≥40	345 (88.5)	45 (11.5)	1.18 (0.60–2.32)	0.621
Ordinate (*n* = 450)				
Monkhood ≤ 1 year	46 (90.2)	5 (9.8)	1	
Monkhood > 1 year	354 (88.7)	45 (11.3)	1.17 (0.44–3.10)	0.753
Secular education (*n* = 476)				
≤Primary education	195 (86.3)	31 (13.7)	1	
>Primary education	226 (90.4)	24 (9.6)	0.67 (0.38–1.18)	0.163
Underlying disease (*n* = 454)				
No underlying disease	219 (88.7)	28 (11.3)	1	
Other underlying disease	180 (89.1)	22 (10.9)	0.96 (0.53–1.73)	0.881
Chronic kidney disease with other underlying disease	5 (100)	0 (0)	–	–
Alcohol drinking before monkhood (*n* = 411)				
No	176 (89.8)	20 (10.2)	1	
Yes	188 (87.4)	27 (12.6)	1.26 (0.68–2.33)	0.455
Smoking (*n* = 480)				
No smoking	175 (89.3)	21 (10.7)	1	
Ex- or current smoking	251 (88.4)	33 (11.6)	1.1 (0.61–1.96)	0.758
Education about parasitic infection (*n* = 404)				
Never	189 (87.5)	27 (12.5)	1	
Received	171 (91.0)	17 (9.0)	0.67 (0.37–1.32)	0.268
Anthelmintic drugs used ≥ 1 time/year (*n* = 331)				
No	67 (88.2)	9 (11.8)	1	
Yes	230 (90.2)	25 (9.8)	0.81 (0.36–1.81)	0.608
Offered raw fish dish ≥ 1 type (*n* = 475)				
No	147 (94.8)	8 (5.2)	1	
Yes	271 (84.7)	49 (15.3)	3.32 (1.53–7.20)	0.002 *
Defecation on ground soil (*n* = 376)				
No	199 (87.7)	28 (12.3)	1	
Yes	136 (91.3)	13 (8.7)	0.68 (0.34–1.36)	0.274

* *p*-value of <0.05.

**Table 4:** The factors associated with skin-penetrating helminth infection in monks.

Variables	Skin-Penetrating Helminth Negative	Skin-Penetrating Helminth Positive	OR_crude_ (95% CI)	*p*-Value
	*n* (%)	*n* (%)		
Age (*n* = 511)				
<40	114 (94.2)	7 (5.8)	1	
≥40	298 (76.4)	92 (23.6)	5.02 (2.26–11.17)	<0.001 *
Ordinate (*n* = 450)				
Monkhood ≤ 1 year	47 (92.2)	4 (7.8)	1	
Monkhood > 1 year	312 (78.2)	87 (21.8)	3.28 (1.15–9.34)	0.026 *
Secular education (*n* = 476)				
≤Primary education	166 (73.5)	60 (26.5)	1	
>Primary education	218 (872)	32 (12.8)	0.41 (0.25–0.65)	<0.001 *
Underlying disease (*n* = 454)				
No underlying disease	207 (83.8)	40 (16.2)	1	
Other underlying disease	158 (78.2)	44 (21.8)	1.44 (0.90–2.32)	0.132
Chronic kidney disease with other underlying disease	1 (20)	4 (80)	20.7 (2.54–190.1)	0.007 *
NSAIDs (*n* = 433)				
Never use	288 (81.6)	65 (18.4)	1	
Using	60 (75.0)	20 (25.0)	1.48 (0.83–2.62)	0.182
Illegal combined drugs (*n* = 433)				
Never used	318 (80.7)	76 (19.3)	1	
Using	30 (76.9)	9 (23.1)	1.25 (0.57–2.75)	0.571
Alcohol drinking before monkhood (*n* = 411)				
No	167 (85.2)	29 (14.8)	1	
Yes	172 (80.0)	43 (20.0)	1.44 (0.86–2.41)	0.167
Smoking (*n* = 480)				
No smoking	171 (87.2)	25 (12.8)	1	
Ex- or current smoking	219 (77.1)	65 (22.9)	2.03 (1.23–3.36)	0.007 *
Education of parasitic infection (*n* = 404)				
Never	165 (76.4)	51 (23.6)	1	
Received	164 (87.2)	24 (12.8)	0.47 (0.28–0.80)	0.006 *
Anthelmintic drugs used ≥ 1 time/year (*n* = 331)				
No	63 (82.9)	13 (17.1)	1	
Yes	213 (83.5)	42 (16.5)	0.96 (0.48–1.89)	0.896
Wearing shoes outside (except during alms work) (*n* = 430)				
Do not always wear	94 (79.0)	25 (21.0)	1	
Always wear	253 (81.4)	58 (18.6)	0.86 (0.51–1.46)	0.579
Defecation on ground soil (*n* = 376)				
No	182 (80.2)	45 (19.8)	1	
Yes	119 (79.9)	30 (20.1)	1.02 (0.60–1.71)	0.940

* *p*-value of <0.05.

## Data Availability

The datasets used and/or analyzed during the current study are available from the corresponding author upon reasonable request.
